# Potential Carbohydrate Regulation Mechanism Underlying Starvation-Induced Abscission of Tomato Flower

**DOI:** 10.3390/ijms23041952

**Published:** 2022-02-10

**Authors:** Qiang Li, Lin Chai, Na Tong, Hongjun Yu, Weijie Jiang

**Affiliations:** Institute of Vegetables and Flowers, Chinese Academy of Agricultural Sciences, Beijing 100081, China; liqiang05@caas.cn (Q.L.); chailin19921017@126.com (L.C.); tongna1994@163.com (N.T.)

**Keywords:** flower abscission, carbohydrate shortage, trehalose-6-phosphate signaling, starch mobilization, phytohormone

## Abstract

Tomato flower abscission is a critical agronomic problem directly affecting yield. It often occurs in greenhouses in winter, with the weak light or hazy weather leading to insufficient photosynthates. The importance of carbohydrate availability in flower retention has been illustrated, while relatively little is understood concerning the mechanism of carbohydrate regulation on flower abscission. In the present study, we analyzed the responding pattern of nonstructural carbohydrates (NSC, including total soluble sugars and starch) and the potential sugar signal pathway involved in abscission regulation in tomato flowers under shading condition, and their correlations with flower abscission rate and abscission-related hormones. The results showed that, when plants suffer from short-term photosynthesis deficiency, starch degradation in flower organs acts as a self-protection mechanism, providing a carbon source for flower growth and temporarily alleviating the impact on flower development. Trehalose 6-phosphate (T6P) and sucrose non-fermenting-like kinase (SnRK1) signaling seems to be involved in adapting the metabolism to sugar starvation stress through regulating starch remobilization and crosstalk with IAA, ABA, and ethylene in flowers. However, a continuous limitation of assimilating supply imposed starch depletion in flowers, which caused flower abscission.

## 1. Introduction

In flowering crops, florescence is the initiation of reproductive growth which determines fruit set and the future yield formation directly. Carbohydrates produced from CO_2_ fixation via photosynthesis in mature leaves (source organs) play significant roles in flowering [1]. As developing flowers are strong sinks for sugars, there will be a distinct allocation shift to floral organs, and this allocation strategy will affect the flowers’ fate. These crops are particularly sensitive to carbohydrate fluctuation during flowering. Any perturbation of the sugar metabolism and supply from source to sink (floral organ) leads to gametophyte abortion, reducing the success of fertilization and increasing the flower abscission rate [2].

Carbohydrate, as a metabolic resource for intermediary metabolism, is related to flower quality and initial ovary growth and retention [3,4,5]. Starch synthesis in rice and maize flowers is essential for mature pollen and flower retention [6,7]. In pepper flowers, defoliation and shading decreased the carbohydrate accumulation in flowers and subsequently caused flower abscission [8,9]. In addition to providing a metabolic resource, sugar acts as a signaling molecule coordinating developmental programs with available carbohydrate via the translation of nutrient status to transcriptional regulation and modulating early reproductive processes [10,11]. However, whether sugar signaling is involved in regulating flower abscission is currently unknown. Trehalose 6-phosphate synthase (TPS) genes showed a different expression pattern in abscising apple fruitlets; however, this has not yet been further investigated [12].

Hormones in reproductive organs have been found to fluctuate in response to carbon availability. As reported in citrus, carbon shortage induced gibberellins (Gas) reduction, and abscisic acid (ABA) and 1-aminocyclopropane-1-carboxylic acid (ACC) increases concomitantly with increased fruitlet abscission [13,14]. Botton et al. [15] and Eccher et al. [16] also found that alterations in the supply of assimilate caused by nutritional stress were translated at fruit level through the crosstalk between sugars and hormones signaling, leading to fruitlet fall off; however, relatively little is understood concerning the complex interactive regulatory mechanism, especially on flower abscission.

Control of flower organ abscission is an important agricultural concern because of its substantial effect on crop yield and quality. In greenhouse, tomato flower abscission often occurs in winter as the weak light or hazy weather leads to weakened photosynthesis. In view of the important role of carbohydrates in flower organ retention and the obscure regulatory mechanism, we sought to explore the mechanism on how to regulate flower abscission through the cooperation of carbohydrate metabolism with hormones in plants suffering starvation stress. Here, we analyzed the responding pattern of nonstructural carbohydrate (NSC, including soluble sugars and starch), the potential sugar signaling pathway, and phytohormone signaling in the tomato flowers under insufficient carbon supply caused by shading. The correlations between flower abscission rate and the response of sugars and hormones in flowers to shading treatment were analyzed to figure out the interactive relationship playing a regulatory role in shading-induced flower abscission. This work will improve our understanding on how flowers respond to carbon starvation and provide fundamental information required for developing horticultural strategies to decrease abscission.

## 2. Results

### 2.1. Physiological Characteristic

**Figure 1:**
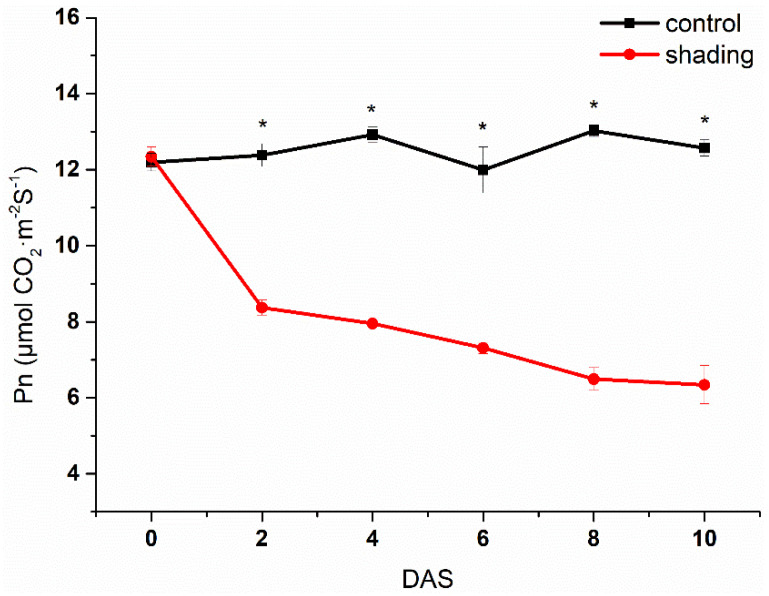
Net photosynthetic rate (Pn) of tomato plants under normal (control) and shading conditions. One–way analysis of variance ANOVA with Duncan’s multiple range test; the values are means ± SD (*n* = 3), * *p* < 0.05.

**Figure 2:**
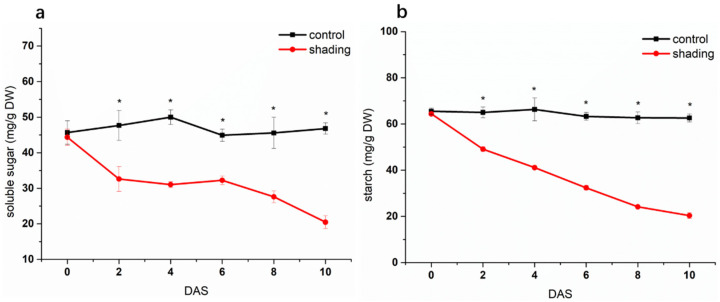
Effect of shading on the content of soluble sugars (**a**) and starch (**b**) in tomato leaves. One–way analysis of variance ANOVA with Duncan’s multiple range test, the values are means ± SD (*n*= 3), * *p* < 0.05.

**Figure 3:**
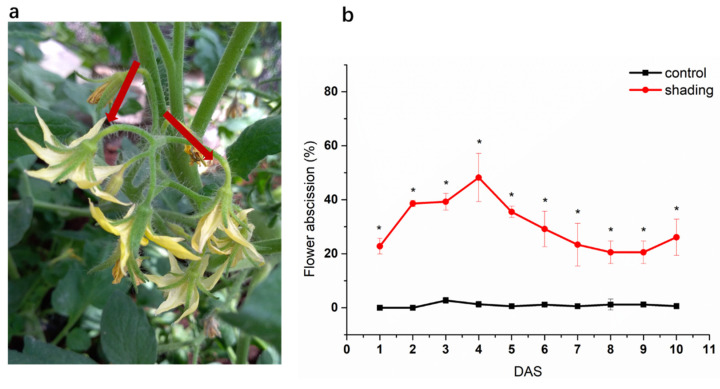
Effect of shading on flower pedicel (**a**) and the abscission rate (**b**). The red arrows in (**a**) mean the distal side of flower pedicels. One–way analysis of variance ANOVA with Duncan’s multiple range test, the values are means ± SD (*n* = 3), * *p* < 0.05.

The source leaves of shaded plants exhibited a significant lower net photosynthetic rate (Pn) when compared to control plants under the normal sunlight condition (Figure 1). The content of non-structural carbohydrate (total soluble sugars and starch) decreased significantly in shaded leaves (Figure 2). With the increase of shading time, the distal side of flower pedicels (the region between the abscission zone and the flower) gradually turned yellow and, finally, the corresponding flowers abscissed (Figure 3a). As shown in Figure 3b, the flower abscission rate in control plants maintained a relatively stable level of less than 5% during the whole experimental period, while it showed a sustainable increase in shaded plants, reaching its maximum peak (50 %) on the fourth day after shading (DAS), before gradually decreasing.

### 2.2. Effect of Shading on Carbohydrate State and Trehalose 6-Phosphate Signaling in Tomato Flowers

#### 2.2.1. Soluble Sugars and Starch

**Figure 4:**
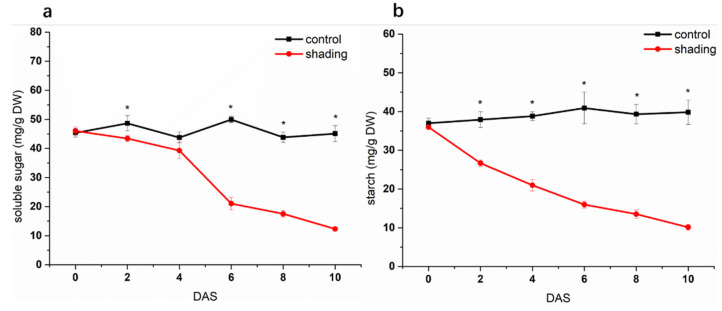
Effect of shading on the content of soluble sugars (**a**) and starch (**b**) in tomato flowers. One–way analysis of variance ANOVA with Duncan’s multiple range test, the values are means ± SD (*n* = 3), * *p* < 0.05.

Under shading conditions, total soluble sugars in tomato flowers remained constant from 0 to 4 DAS, then declined and showed lower levels than that of control (Figure 4a). Starch in flowers decreased slightly at the beginning of shading, then showed a continuous decrease, which caused significant differences between control and treatment (Figure 4b). Compared to control, shading treatment reduced carbohydrate supply for flower organs, which might have led to greater starch degradation in flowers to maintain sugar homeostasis. To further verify the response of starch to carbon starvation, we exposed the whole plant to 4 h extended night to reduce the carbohydrate supply from source leaves. As Supplementary Figure S1 shows, flower starch was obviously lower in plants exposed to an extended night, while the total soluble sugars did not vary between control and the extended night sample. Thus, we proposed that plants will strive to meet the demand from flower organs for sugars through regulating starch metabolism in flowers when photosynthetic carbon is in short supply.

**Figure 5:**
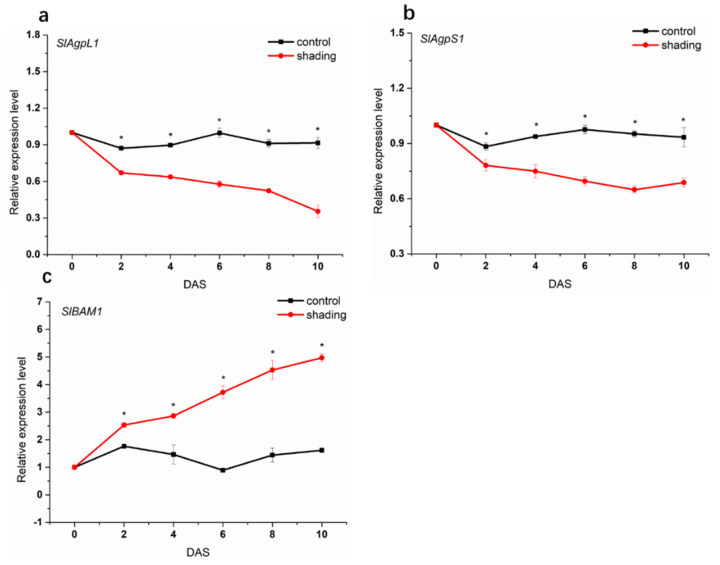
Effect of shading on the transcript levels of starch metabolism enzyme in tomato flowers. (**a**) *AgpL1*, the large subunit–encoding gene of AGPase. (**b**) *AgpS1*, the small subunit–encoding gene of AGPase. (**c**) *BAM1*, β–amylase gene. One–way analysis of variance ANOVA with Duncan’s multiple range test, the values are means ± SD (*n* = 3), * *p* < 0.05.

ADP-glucose pyrophosphorylase (AGPase) and β-amylase play important roles in starch synthesis and degradation, respectively [17]. Here, we examined their transcript level in tomato flowers during shading to elucidate the starch metabolism mechanism in response to carbon starvation. Figure 5 shows that AGPase genes (the large subunit-encoding gene *AgpL1* and small subunit-encoding gene *AgpS1*) were down-regulated in flowers after shading for 2 days and exhibited significantly lower levels than those of control. However, *BAM1*, one of the β-amylase genes, was up-regulated in shaded flowers compared with control.

#### 2.2.2. Trehalose 6-Phosphate Signaling

**Figure 6:**
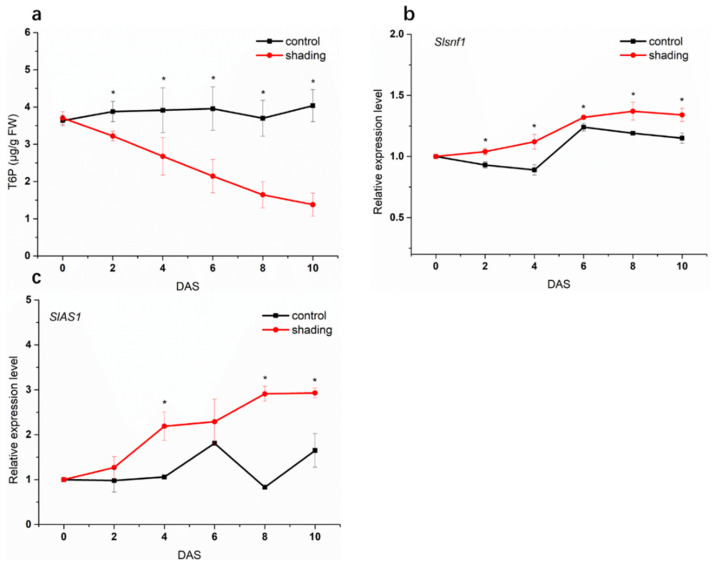
Effect of shading on T6P/SnRK1 signaling pathway in tomato flowers. (**a**) T6P content. (**b**,**c**) the transcript levels of SnRK1 subunit gene *Snf1* and SnRK1 marker gene *AS1*. One–way analysis of variance ANOVA with Duncan’s multiple range test, the values are means ± SD (*n* = 3), * *p* < 0.05.

Trehalose 6-phosphate (T6P) acts as a signal of carbon availability and greatly influences sink organ development [18]. In the present study, T6P content in shaded flowers was lower than in control and showed an obvious decrease trend with the increase of shading time (Figure 6a). T6P is not a growth signal per se, but it links nutrient stress to a number of response pathways by inhibiting the activity of SnRK1 (SNF1-related protein kinase), which acts as a central regulator of caron metabolism [19,20,21,22]. As shown in Figure 6b, SnRK1 subunit SNF1 gene expression was up-regulated in shaded tomato flowers. Moreover, the SnRK1-induced marker gene *AS1* (Asparagine Synthase 1), which can indicate the SnRK1 activity, showed an increasing transcript level after shading for two days (Figure 6c).

### 2.3. The Response of Phytohormone Signaling in Tomato Flowers to Weak Light

**Figure 7:**
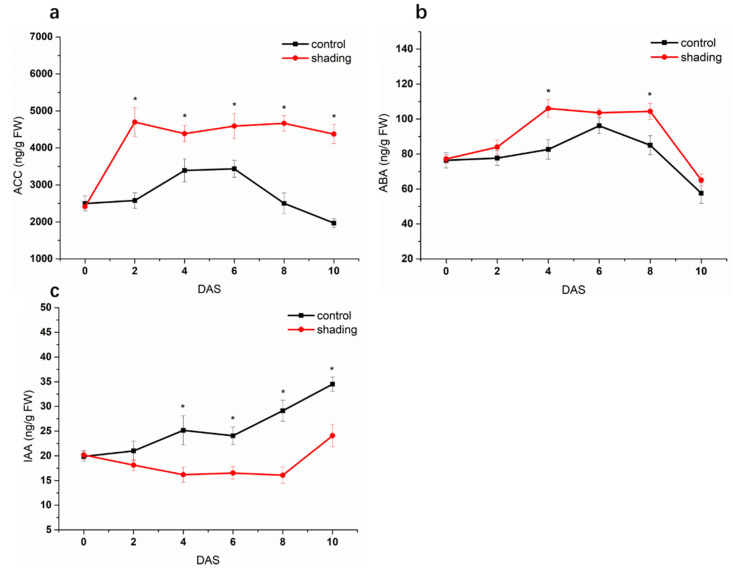
Effect of shading on the content of phytohormones in tomato flowers. (**a**) ACC. (**b**) ABA. (**c**) IAA. One–way analysis of variance ANOVA with Duncan’s multiple range test, the values are means ± SD (*n* = 3), * *p* < 0.05.

To explore the response of phytohormones to weak light in tomato flowers, we analyzed the phytohormone profile in tomato flowers under shading condition. As shown in Figure 7, the content of ACC (1-aminocyclopropane-1-carboxylic acid, the ethylene precursor) was significantly enhanced in the shaded samples, with the highest level occurring at 4 DAS. ABA also presented higher levels in shaded flowers compared with control and showed a biphasic dynamic: the first peak occurred at 2 DAS, and the second occurred at 6 DAS; a progressive decrease was observed thereafter. However, shading significantly decreased auxin levels in flowers.

**Figure 8:**
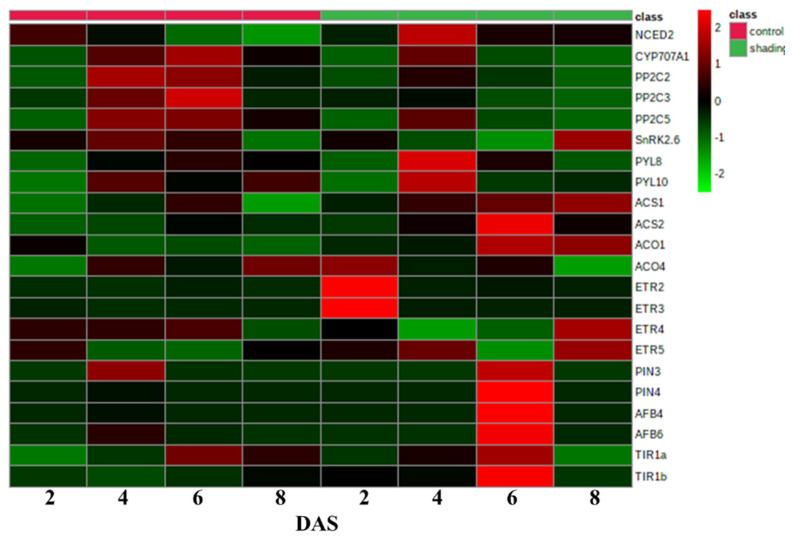
Heat map for the expression of phytohormone signaling genes in tomato flowers under normal and shading conditions. It was generated based on the fold change of gene expression levels in control and shaded flowers at the 2nd, 4th, 6th, 8th, and 10th day after treatment when compared with the sample at 0 days after treatment. The values are means of three biological replicates with three technical replicates.

To gain a global view of the transcriptional regulation mechanisms associated with the fluctuation of hormones in shaded flowers, we focused on the expression patterns of genes involved in hormone metabolism and their signaling pathways. The expression profiles were examined in both control and shaded tomato flowers (Figure 8). Compared to control flowers, shading upregulated ABA biosynthetic gene *NCED2* and down-regulated ABA catabolism gene *CYP707A1*. The core components in the ABA signal transduction pathway, including PYR/PYL/RCAR ABA receptors (SlPYLs), protein phosphatases 2C (PP2Cs), and SNF1-related protein kinase 2 (SnRK2), were also affected by shading at the transcript level. Gene expression of *PP2C*, which is involved in the negative regulation of ABA signal transduction, was suppressed after shading treatment, while *PYLs* and *SnRK2* were generally up-regulated in shaded flowers. The ethylene biosynthetic and signal transduction related genes in flowers showed different sensitivities to shading and were generally expressed higher than in control. The transcriptional levels of auxin efflux carrier PIN-FORMED (PIN) and auxin receptor (TIR1/AFB) were relatively steady in control flowers but were dramatically upregulated after six days of shading.

### 2.4. Carbohydrate and Phytohormones in Relation to Tomato Flower Abscission Affected by Weak Light

**Figure 9:**
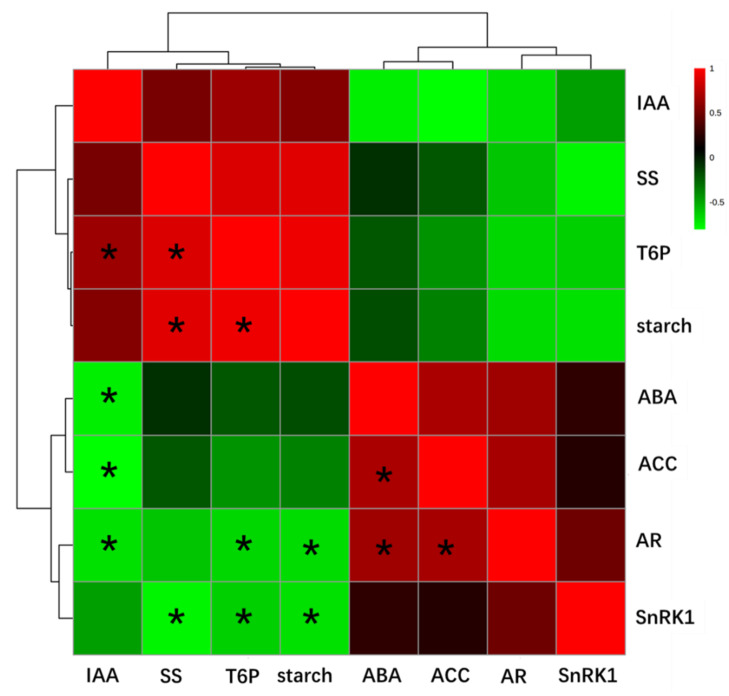
Correlation analysis between tomato flower abscission rate and carbohydrate state and phytohormones (* *p* < 0.05). SS, slouble sugars; AR, abscission rate.

To further investigate the potential regulators in the flower drop process, the relationships among carbohydrate state, T6P signaling, and hormone signaling in tomato flowers under shading condition and the flower abscission rate were analyzed. As shown in Figure 9, the flower abscission rate was negatively correlated with dynamics of T6P, starch, and IAA in tomato flowers during the shading process, while it was positively correlated with the content of ABA and ACC. Moreover, T6P correlated positively with starch and IAA content, while IAA was negatively correlated with ABA and ACC. Finally, ABA and ACC showed a positive correlation.

Despite the significance of carbohydrate content to tomato flower retention, a question remains as to whether abscised flowers induced by any other situation (such as physiological abscission) also have lower sugar levels. To this end, we compared the contents of carbohydrate and hormones in abscised flowers and on-plant flower under normal and shading conditions respectively (Table 1). After shading for six days, the abscised flower contained lower levels of carbohydrate than did flowers remaining on plants, while the contents of ABA and ACC were lower in the on-plant flowers. Similarly, the hormone levels were higher in abscised flowers in plants grown under normal conditions, but no significant differences were found in soluble sugars and starch content between abscised flowers and on-plant flowers.

## 3. Discussion

Photosynthesis is closely connected with the capacity of plants to produce assimilate, which is a crucial factor for vegetable growth. Assimilate partitioning into reproductive organs is critical in flower retention and fruit set [1,23,24,25]. The distinct susceptibility of pepper cultivars to flower abscission might relate to their different power to produce sugars and accumulate starch [24]. Carbohydrate shortage was shown to lead to dramatic fruitlet abscission in lychee [23]. In the present study, shading affected light interception and impaired photosynthesis, which caused a decrease in available carbohydrate for the developing flowers and increased the rate of flower abscission.

T6P is considered to be a signal of carbon availability, and its fluctuation can reflect the carbohydrate state in plants [7,8,9,26]. Here, we found lower levels of T6P in flowers in response to shading, which implied a decrease in carbon accumulation in flower organs under weak light. The analysis on nonstructural carbohydrates (soluble sugars and starch) in shaded flowers supported this idea. Some studies have shown that T6P can regulate starch mobilization in source leaves to balance sucrose supply with demand from sink organs [27,28]. In our study, the starch degradation process became active in shaded flower organs. There was a strong and positive correlation between T6P and starch, indicating that T6P might also regulate starch mobilization in reproduction organs to cope with the short supply of photoassimilates.

The major regulating function of T6P is mediated through inhibiting SnRK1 activity [19,20]. A lower T6P level elicits more active SnRK1, which regulates the expression of serious genes involved in catabolic rather than anabolic processes [20]. In our study, the expression level of SnRK1 was up-regulated in shaded tomato flowers, and a similar pattern of regulation was also found in the asparagine synthase 1 gene (*AS1*), which has previously been used as a reporter of SnRK1 activity [6]. It implies that SnRK1 was activated in flowers under shading conditions, which makes sense with the decline of T6P. The regulation of T6P/SnRK1 in flowers is important for suspending growth when carbohydrate is in short supply, thus preventing starvation and withering.

Continuous limitation of assimilate supply to flowers/fruitlets imposed an energy deficit which caused flower/fruit abscission, as observed in this study and previous studies [15,29]. This process may be mediated by phytohormones, which can accelerate or inhibit flower abscission [3,4,30]. In citrus trees, defoliation at anthesis triggered a shortage of carbohydrate and increased ABA and ACC levels sequentially in fruitlets before their abscission. ABA was proposed to act as a modulator of ACC levels, and then of ethylene biosynthesis [31]. In the present study, the increase of ABA biosynthetic and signaling genes (*NCED2*, *PYL8*, *PYL10*) in shaded flowers responded quicker than that of ACC biosynthetic gene (*ACS1*, *ACS2*), while a faster reaction was shown for ethylene biosynthesis and signal transduction genes (*ACO4*, *ETR2*, *ETR3*). It has been generally accepted that auxin and ethylene regulate the onset of abscission in an antagonistic fashion. Organ abscission is inhibited by keeping a continuous polar flow of auxin through the abscission zone to make it insensitive to ethylene [32,33,34]. Shading decreased IAA in tomato flowers, indicating its involvement in crosstalk with ABA and ethylene to regulate the abscission of sink organs induced by carbon shortage.

The interconnected relationship between hormones and sugars in regulating plant growth and development has been accepted, while information on the detailed mechanisms is still limited [35]. Matthew et al. speculated that T6P affected the expression of auxin signaling genes probably in a SnRK1-independent manner [35,36]. In the present study, T6P content correlated with IAA content and abscission rate positively, while no significant correlation existed between SnRK1 and hormones. Thus, we speculated that T6P signaling, at least in part, mediated tomato flower abscission through its effect on IAA level and signaling. Moreover, a previous study reported that ABA is a key signaling molecule regulating the SnRK1-dependent sugars demand signaling pathway under abiotic stress [37]. ABA positively regulates SnRK1 signaling, which is involved in starch degradation and energy deficit responses to promote plant survival during energy stress by inhibiting ABA signaling repressor PP2Cs [38]. Here, both ABA content and SnRK1-induced marker gene expression were up-regulated in shaded flowers, suggesting ABA is probably involved in SnRK1 signaling in flower organs to promote starch remobilization to withstand starvation stress.

When photosynthates supply was insufficient, the phytohormones promoting flower abscission were significantly elevated in tomato flowers. Not all flowers fell at once, as it mainly depended on the level of carbohydrate stored in each flower. Flowers with greater starch accumulation can survive short-term carbon starvation through its degradation, but flowers with insufficient starch storage will fall off earlier under the action of hormones. When flowers fell under the normal growth condition, the hormones in the abscised flowers fluctuated greatly, but the sugars did not change significantly. Similarly, Antonio et al. (2020) found the physiological flower abscission in avocado did not seem to depend on competition for photoassimilates [39].

## 4. Materials and Methods

### 4.1. Plant Material and Growth Conditions

Tomato seeds, *Solanum lycopersicum* L. *cv.* Monkey maker, were germinated on a culture dish at 28 °C in dark. The germinated seeds were sown in seedling plugs and the uniform seedlings with four expanded leaves (32 days after germination) were transplanted into 8 L pots filled with commercial cultivation substrate (Shandong Shangdao Biotechnology Co., LTD, Jinan, Shandong, China). All plants were grown in the greenhouse at the Institute of Vegetables and Flower, Chinese Academy of Agricultural Sciences (Beijing, China, 40.11° N 116.16° E). The changing environmental parameters in the greenhouse were supervised by environmental monitoring probes (Beijing Wisteria Wire Technology Co. LTD, Beijing, China) and are shown in Supplementary Table S1.

The plants were topped when their third truss had flower buds. To avoid any effects of fruit, the first truss was removed. The flowers in the second and third truss were the target samples in this experiment. For consistency, 6 flowers per truss were retained. After topping and removing the redundant flowers and fruits mentioned above, 180 uniform plants were divided into three blocks and used for this experiment. The plants from each block were divided into two parts, one was for control (no shading, 30 plants), the other was for shading (about 70% polypropylene shade over the entire plant for 10 consecutive days, involving 30 plants). The duration of the greenhouse experiment was 10 days. Open-flowers from the corresponding plants (25 plants) were collected at 0, 2nd, 4th, 6th, 8th and 10th day after treatment. The remaining five plants were used for calculating the flower abscission rate, and the abscissed flowers were also collected every day. Three biological replicates were conducted in the blocks independently. All samples were immediately frozen in liquid nitrogen and stored at −80 °C.

### 4.2. Photosynthesis Measurement

The net photosynthesis rate for control and shading treatment plants in each block was measured with the portable photosynthesis system (Li-6400XT, Li-COR, Inc., Lincoln, NE, USA) independently. Six plants in each block were chosen randomly, and their fourth leaves counted from top to bottom were measured at the 2nd, 4th, 6th, 8th, and 10th day after treatment. The setting parameters were as follows: photosynthetic photon flux density, 800 μmol·m^−2^·s^−1^; CO_2_ concentration was matched according to air condition.

### 4.3. Carbohydrate Analysis

According to Liu et al. [40], soluble sugars and starch in tomato leaves and flowers were extracted and measured. Briefly, 0.02–0.03 g ground plant tissue was homogenized in 5 mL 80% (*v*/*v*) ethanol, 80 °C water bath for 30 min, and centrifuged at 8000 rpm for 10 min. The supernatant was collected into a 50 mL centrifuge tube. The residues were extracted twice according to the above steps. An amount of 1 mL supernatant was mixed with 5 mL anthrone sulfuric acid. After vortexing, the mixture was incubated in boiling water bath for 10 min and the A_625_ was read. The residues were used for starch content determination. Double-distilled water (5 mL) was mixed with the residues and incubated in a boiling water bath for 15 min. After cooling, 2 mL perchloric acid was added into the mixture and it was vortexed for 20 min. The supernatant was collected by centrifugation at 8000 rpm for 10 min. The anthranone sulfuric acid method mentioned above was used to measure starch content.

Trehalose 6-phosphate (T6P) was extracted and measured as described by Sastre Toraño et al. [41]. Briefly, 100 mg flower tissue was extracted with 500 μL of chloroform/ acetonitrile mixture by shaking for 2 h at −10 °C. The aqueous phase containing acetonitrile was evaporated to dryness, reconstituted in water, and further cleaned up by solid-phase extraction. The phosphordisaccharide was separated with a freshly prepared 2% (*v*/*v*) formic acid solution in methanol and injected directly into the UPLC system. Disaccharide phosphates were separated on an AQUITY UPLC BEH C18 column (2.1 × 50 mm) with 1.7 μm particles from Waters (Shanghai, China) at 30 °C. The mobile phase at a flow rate of 0.2 mL/min included A (acetonitrile) and B (water). The optimized gradient conditions were as follows: solvent A maintained at 100% for 4 min, then changed from 100% to 60% in 0.5 min, and held at 60% for 3.5 min, then changed from 60% to 0 in 2 min, returning from 0 to 100% in 3 min, and, finally, maintained at 100% for 2 min. Detection was performed with an ion trap from Agilent Technologies equipped with an ESI source.

### 4.4. RNA Extraction and Quantitative Real Time-PCR (RT-qPCR)

Total RNA of tomato flowers that had been finely ground in liquid nitrogen was extracted using Relatthe TRIzol reagent (Invitrogen, Carlsbad, CA, USA). The genomic DNA was digested using a Recombinant RNase-free DNase I kit (Takara, Kusatsu, Shiga, Japan). RNA content and quality were assessed with an NanoDrop ND-2000 Photospectrometer, and RNA integrity was confirmed by electrophoresis on 1% agarose gel. cDNA was synthesized with 5 μg of total RNA using the M-MLV reverse transcriptase (Promega, Madison, WI, USA).

Relative expression levels of the genes were analyzed by iQ™5 Multicolor Real-time PCR Detection system (Bio-Rad) with a SYBR Premix Ex Taq kit (Takara). The specific primers are provided in Supplementary Table S2 and *SlActin* was used as an internal control to normalize all data. The sample from before shading (0 day after treatment) was used as reference to calculate the relative quantification with the method described by Livak et al. [42].

### 4.5. Hormone Extraction and Analysis

An aliquot of flower tissue (1 g) was ground to a fine powder in liquid nitrogen and extracted with 5 mL of cold extraction solution (−20 °C, methanol/water/formic acid, 15/4/1, *v*/*v*/*v*, pH 2.5). After 10 h extraction at −20 °C in the dark, the solids were separated by centrifugation (14,000 rpm, 15 min) and re-extracted in an additional 5 mL of the same extractor for 30 min. The supernatants were pooled and passed through a Sep-Park Plus C18 cartridge (SepPark, Waters, Massachusetts, USA) and evaporated to dryness. The residue was resuspended in 5 mL of 1 M formic acid and loaded on an Oasis MCX mixed column pre-conditioned with 5 mL of methanol followed by 5 mL of 1 M formic acid. The IAA and ABA on the column were washed and eluted according to the method of Dobrev and Kaminek [43]. IAA and ABA in the same elution fraction were then evaporated at 40 °C under vacuum. The residue was dissolved in a water/acetonitrile/formic acid (94.9/5/0.1, *v*/*v*/*v*) mixture for HPLC/MS system. Quantitative analysis of IAA and ABA was performed by Agilent 1200 Series HPLC-MSD trap VL equipped with ESI source, essentially as described by Alfonso et al. [44].

ACC content was extracted according to Mou et al. [45]. One gram of each powdered sample was extracted on ice with 10 mL 95% aqueous ethanol (*v*/*v*), then separated by centrifugation at 10,000 rpm for 15 min at 4 °C. The supernatant was evaporated to dryness under vacuum at 40 °C, and the residue was dissolved in 5 mL distilled water. Following oxidative conversion, the ACC content was determined as ethylene by gas chromatography (SHIMADZU, GC-2014C PF, Tokyo, Japan), as Lizada and Yang described [46].

### 4.6. Statistical Analysis

The experiments in this study were repeated three times, and the data were analyzed using the SPSS 22.0. Statistically significant differences among means were determined by one-way analysis of variance ANOVA with Duncan’s multiple-range test at *p* < 0.05. Heat map generation and correlation analysis were carried out using the MetaboAnalyst 4.0 on-line software (https://www.metaboanalyst.ca/MetaboAnalyst/faces/home.xhtml) accessed on 15 January 2021 [47].

## 5. Conclusions

**Figure 10:**
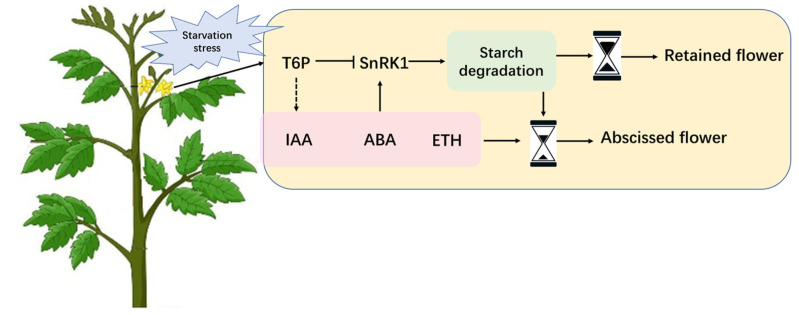
Hypothetical model for tomato flower abscission under carbon starvation. Sugar starvation stress induces starch degradation in flowers to strive to be survival. T6P and SnRK1 signaling pathways will be involved in this process to regulate starch remobilization in flowers. Although the fluctuations of phytohormones are in favor of flower abscission, the fate of the flower is largely dependent on their own starch reserves under carbon stress. It looks like a sand clock, more reserves in flowers will have a substantial chance at survival, and vice versa.

In tomato florescence, a decrease in supply of carbohydrate to flowers elicits a higher abscission rate. T6P and SnRK1 signaling seem to be involved in adapting metabolism to sugar starvation stress through regulating starch remobilization in flowers. Combining these, a model was devised for tomato flower abscission caused by insufficient carbon supply (Figure 10). As abiotic stresses lead to a shortage of carbohydrate allocation, flower organs attempt to manage it by using their own carbohydrate reserves, during which process the T6P and SnRK1 signaling pathways regulate starch remobilization in the flowers. On the other hand, the fluctuation of T6P triggers the IAA signaling pathway, which engages in crosstalk with ABA and ethylene signaling to promote flower abscission. In this case, the fate of flowers is closely related to their starch reserves. Flowers with insufficient reserves or under sustained stress will prefer to fall off with the promotion of hormones. Finally, manipulating the carbohydrate level in flowers by genetic engineering or chemical intervention to cope with abscission induced by carbon starvation might be a potential agronomic strategy.

## Figures and Tables

**Table 1:** Comparison of carbohydrate and phytohormones between abscissed flowers and on-plant flowers under normal and shading conditions.

		Carbohydrate (mg/g DW)		Phytohormone (ng/g FW)	
		Soluble Sugars	Starch	ABA	ACC
control	abscissed flower	51.96a	41.8a	105.38b	4821.55a
	on-plant flower	50.75a	41.22a	93.45d	3526.82c
shading	abscissed flower	9.57c	8.34c	108.43a	4782.42a
	on-plant flower	19.76b	15.88b	102.68c	4523.24b

Values are means of three replicates; different letters in the same column indicate significant difference (*p* < 0.05).

## Data Availability

Not applicable.

## Supplementary Materials

The following supporting information can be downloaded at: https://www.mdpi.com/article/10.3390/ijms23041952/s1.
