# Supplementary material for: Potential Carbohydrate Regulation Mechanism Underlying Starvation-Induced Abscission of Tomato Flower
Source: Int J Mol Sci. 2022 Feb 10;23(4):1952. doi: 10.3390/ijms23041952 (PMC8876634; doi:10.3390/ijms23041952)
Supplement: Supplementary file 1 [file ijms-23-01952-s001.zip › ijms-1568062-supplementary.pdf]

Table S1. The environmental parameters in the greenhouse

Date	Temp (℃)		RH (%)		Lm (lx)	CO_2(ppm)		Substrate Temp(℃)	
	Max	Min	Max	Min	Max	Max	Min	Max	Min
2020/11/21	23.14	14.46	61.50	33.25	11630	618	417	20.81	14.81
2020/11/22	25.11	13.47	53.66	36.18	12310	705	417	18.37	13.06
2020/11/23	29.00	14.55	56.17	41.57	13960	741	417	20.50	14.00
2020/11/24	29.62	16.30	57.66	45.92	11260	776	419	22.06	15.62
2020/11/25	30.28	17.66	58.28	46.78	11340	865	417	23.18	17.00
2020/11/26	28.13	17.86	54.09	23.16	11770	739	417	20.56	17.25
2020/11/27	27.18	17.26	61.08	50.85	10260	794	490	21.37	16.75
2020/11/28	26.20	19.00	63.38	53.39	8690	813	507	21.87	18.31
2020/11/29	20.92	17.92	63.51	50.18	4490	812	417	19.06	17.62
2020/11/30	25.86	15.24	53.89	39.96	14370	610	415	19.31	15.43

Table S2. Primers used for qPCR

Gene Name	ACCESSION	Sense primers	Antisense primers
Actin	NM_001308447.1	ATCCCAAGGCCAACAGAGAG	CGACCGCTAGCATACAGAGA
NCED2	NM_001375893.1	TGGTTTTCATGGGACATTCATTAGC	ATCTCCCTTCTCAACTCCCTATTCC
CYP707A1	NM_001247588.2	CCCAGAGTTCTTTCCTGATCCACAA	GAATGCCACTACCAGATCCTACCAC
PP2C2	NM_001247763.2	CAGTGATGGATTATGGGACGTGGTA	CCTAGCCAAGGCTAATTTCGTCAA
PP2C3	XM_015224583.2	TGGCAGAGTAATCTACTGGGACGG	TGAGACTACGTCCCATAATCCGTCA
PP2C5	XM_015214354.2	GTGTATTTGGCGTTCTTGCAATGTC	CAGGCAGAGGGTTAGTCCCGTTC
SnRK2.6	NM_001247424.2	CAGCAATGACTTTGCTGTTTATGTCTG	CGTGAATCCGAATTAGACGGATACC
PYL8	XM_004234127.4	CTAGTGTACATGGATTCGAACGCCA	AGATCTCACCGTTACCACCAAGAGC
PYL10	XM_015215524.2	CACCCAGAGGTGATTGATGGAAGA	TCAATGGGTTCTGTCCTGTCTTGC
ACS1	NM_001279342.3	GAAAGAGTTGTTATGGCTGGTGG	AATGCTGGGTAGTATGGTGAAGG
ACS2	NM_001247249.3	AGAGTTGTTATGGTTGGTGGTG	GCTGGGTAGTATGGTGAAGGT
ACO1	NM_001247095.2	AGGAGGCATCATACTTCTGTTC	ACAATAGAGTGGCGCATGGGA
ACO4	NM_001246938.2	AAAGAAATGGTGGCAAGTAAAGG	TGTATTCATCATCCAGGTCAGG
ETR2	NM_001247224.2	GGATTTCCCCAAGTTGTTCTG	CCCTTCCCGACACCTTCAC
ETR3	NM_001246965.2	AAGTTGCTGTGGCGTTGTCC	CGATGGCTCATAACCGTCTG
ETR4	NM_001247276.2	TCGTCCTTTTGGATCTTCACC	CGATACCTGGCAATAGCACTG
ETR5	NM_001247283.2	CGTAAGTTCCGCAGCCGTAG	TTCATCAGCCAAGCCTTGTAG
PIN3	NM_001247248.2	AACAATTCCCAGAAACAGCA	GTTTCCCATCTCGCCCTA
PIN4	NM_001247255.2	TCAGAGTTCCCCGAGGTTC	TGAGGTTTTGTGCTTTTAGGC
AFB4	NM_001247793.1	ACGGTCACTGATGAACCAGA	TGCAGGCAGATATTCAGGAG
AFB6	XM_010318372.3	GAATCAATGCGGTGTCTTTG	GTCATCGCTGTGCTCATCTT
TIR1a	NM_001247744.2	GGTGATAGCGATCTGGGACT	CTCGAGCTTTGCAGCATTAG
TIR1b	XM_006362921.2	GGCTCTGTTGGCTATTGCTGC	CATTGCTATATGGAGTCGATG
AS1	NM_001319849.2	GCACGGCTAAAGCAATCGAG	ATCGGGGTGTCCAAATTCCC
Snf1	XM_015215646.2	CTCCTGAGGTTGTATCTGGGA	TGGTATCAAATCCCTAGCTAAAGG
BAM1	NM_001247627.2	GAAGGAAGGTGGTGGATGGG	TTGGGCGATGGGAAGGTAAC
GWD	NM_001247476.2	TGAGGTGGGAAAGGAAGGGA	CTTGGGCAAGGTCATCAGGT
PWD	XM_004247960.4	TTATCCCCGGCACTCTACCA	GTCATCGTCATCGCAGGTCA
AgpL-1	NM_001246989.2	AGATCGCGCTTAGACTGTGG	TCGGTCTGCCTCTTGAACAC
AgpS-1	NM_001247767.3	TGTTCAAGAAGCGGCTAGGG	TTCGGCACGTTTGTACCTCA

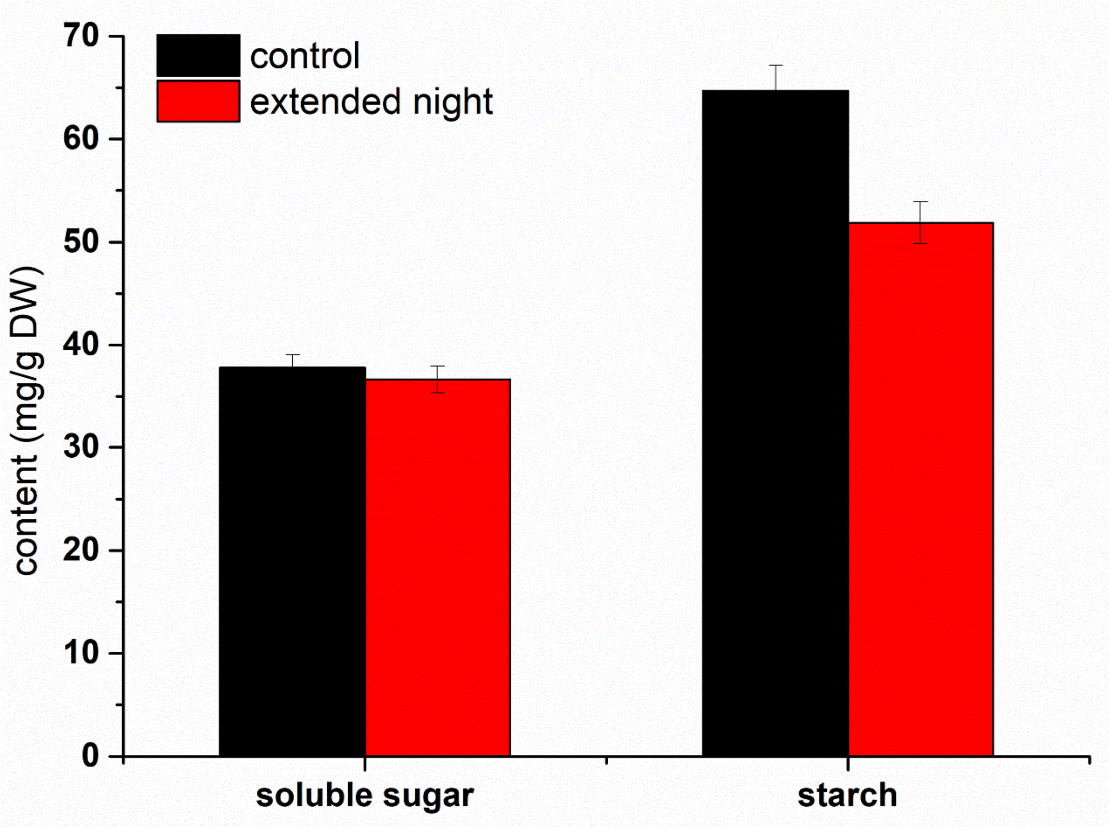

Figure S1. Effect of extended night on soluble sugar and starch in tomato flowers
